# Maternal complications and risk factors associated with assisted vaginal delivery

**DOI:** 10.1186/s12884-023-06080-9

**Published:** 2023-10-26

**Authors:** Saifon Chawanpaiboon, Vitaya Titapant, Julaporn Pooliam

**Affiliations:** 1 Division of Maternal-Fetal Medicine, Department of Obstetrics and Gynaecology, Faculty of Medicine Siriraj Hospital, Mahidol University, Bangkok, 10700 Thailand; 2 Clinical Epidemiological Unit, Office for Research and Development, Faculty of Medicine Siriraj Hospital, Mahidol University, Bangkok, 10700 Thailand

**Keywords:** Assisted, Forceps, Maternal complication, Vacuum, Vaginal delivery

## Abstract

**Objective:**

This study aimed to elucidate the maternal complications and risk factors linked with assisted vaginal delivery.

**Methods:**

We conducted a retrospective, descriptive analysis of hospital records, identifying 3500 cases of vaginal delivery between 2020 and 2022. Data encompassing demographics, complications from the vaginal delivery including post-partum haemorrhage, birth passage injuries, puerperal infection and other pertinent details were documented. Various critical factors, including the duration of the second stage of labor, maternal anemia, underlying maternal health conditions such as diabetes mellitus and hypertension, neonatal birth weight, maternal weight, the expertise of the attending surgeon, and the timing of deliveries were considered.

**Results:**

The rates for assisted vacuum and forceps delivery were 6.0% (211/3500 cases) and 0.3% (12/3500), respectively. Postpartum haemorrhage emerged as the predominant complication in vaginal deliveries, with a rate of 7.3% (256/3500; *P* < 0.001). Notably, postpartum haemorrhage had significant associations with gestational diabetes mellitus class A1 (adjusted odds ratio [AOR] 1.46; 95% confidence interval [CI] 1.01–2.11; *P* = 0.045), assisted vaginal delivery (AOR 5.11; 95% CI 1.30–20.1; *P* = 0.020), prolonged second stage of labour (AOR 2.68; 95% CI 1.09–6.58; *P* = 0.032), elevated maternal weight (71.4 ± 12.2 kg; AOR 1.02; 95% CI 1.01–1.03; *P* = 0.003) and neonates being large for their gestational age (AOR 3.02; 95% CI 1.23–7.43; *P* = 0.016).

**Conclusions:**

The primary complication arising from assisted vaginal delivery was postpartum haemorrhage. Associated factors were a prolonged second stage of labour, foetal distress, large-for-gestational-age neonates and elevated maternal weight. Cervical and labial injuries correlated with neonates being large for their gestational age. Notably, puerperal infections were related to maternal anaemia (haematocrit levels < 33%).

**Clinical trial registration:**

Thai Clinical Trials Registry: 20220126004.

## Introduction

Vaginal birth is a natural and physiological process. It encompasses sequential, integrated alterations in the myometrium, decidua and cervix that transpire progressively over days to weeks. This ultimately leads to swift changes over hours, culminating in the expulsion of the products of conception, namely, the foetus and placenta [1]. The vaginal delivery route is preferred and universally accepted and offers minimal harm to both the mother and foetus. This method facilitates fast recovery for the mother, enabling her to initiate breastfeeding promptly.

Vaginal deliveries can be categorised into spontaneous and induced types. A spontaneous vaginal delivery [2] transpires when a pregnant woman undergoes labour without any pharmacological intervention or techniques to induce labour and gives birth without the assistance of forceps or vacuum extraction. Conversely, an induced vaginal delivery involves initiating labour through pharmacological or manual techniques [3].

Assisted vaginal births, primarily employed to hasten the birthing process for the welfare of both mother and infant, can occasionally introduce morbidity. Predominantly, forceps and vacuum instruments are utilised, with the choice often influenced by clinical scenarios, the obstetrician’s preference, experience and instrument availability. Assisted vaginal delivery proves pivotal for mothers facing labour challenges. Techniques such as vacuum extraction and forceps assistance are reserved for specific indications. To mitigate procedural complications for the mother and foetus, these methods must be conducted by experienced or adequately trained obstetricians. Data from the United States in 2017 indicate that 3.1% of all births utilised an assisted vaginal approach [4], with forceps and vacuum deliveries constituting 0.5% and 2.6% of vaginal births, respectively. Notably, the prevalence of assisted vaginal births demonstrated considerable variance across and within the United States [4]. Such disparities suggest that the choice of delivery method often hinges on a clinician’s familiarity and proficiency with the technique [5]. On a broader scale, the United States has witnessed a decline in the national and regional rates of assisted vaginal births [6].

A surge in caesarean section rates correlates with a decline in assisted vaginal deliveries, compounded by an erosion of the requisite obstetrical skills [7]. In exigent scenarios, assisted vaginal delivery can be pivotal for neonatal survival. Inadequate expertise can precipitate complications and unforeseen incidents. This study investigated maternal complications resulting from vaginal deliveries, scrutinising detailed records of such complications experienced by mothers at Siriraj Hospital, Bangkok, Thailand. Concurrently, it explored the risk factors associated with maternal complications.

## Methods

This retrospective investigation was undertaken within the statistical unit of the Department of Obstetrics and Gynaecology, Faculty of Medicine, Siriraj Hospital. Before the research commenced, its protocol was approved by the institution’s Ethics Committee (Si 185/2022) and was registered with the Thai Clinical Trials Registry (20220126004). The authors are grateful to the Faculty of Medicine, Siriraj Hospital, Mahidol University, for its financial support ([IO] R016533036).

In the data collection phase, we obtained records from hospital archives spanning the years 2020 to 2022, identifying a total of 3500 cases involving women who delivered vaginally at Siriraj Hospital. Our inclusion and exclusion criteria were applied to participants based on the completeness of their medical records, excluding those with incomplete data.

During data recording, we diligently documented baseline characteristics, demographic information, relevant laboratory results, underlying medical conditions, details regarding previous vaginal deliveries, and maternal complications. The primary objective of this study is to conduct a comprehensive analysis of maternal complications resulting from vaginal deliveries at Siriraj Hospital.

Our secondary outcomes encompass a thorough exploration of maternal complications, including postpartum hemorrhage, infections, and injuries to the vaginal birth passage. Additionally, we investigate various factors associated with maternal complications, such as maternal weight and the duration of labor during the active phase, among other relevant variables. Furthermore, we assess the influence of attending medical personnel on these complications.

### Factors associated with maternal complications arising from vaginal deliveries

Our study places primary emphasis on maternal complications, specifically postpartum hemorrhage (PPH), birth passage injuries, and puerperal infection. To conduct a thorough and comprehensive analysis of these maternal complications, it is vital to explicitly delineate the contributing factors leading to these adverse outcomes. Significant factors for consideration encompass the duration of the second stage of labor, maternal anemia, underlying maternal health conditions such as diabetes mellitus and hypertension, neonatal birth weight, maternal weight, the expertise of the attending surgeon, and the timing of deliveries. Our analysis will delve deeply into the intricate relationship between assisted vaginal delivery and the incidence of maternal complications and associated risk factors, thus shedding essential light on this critical aspect of our research.

### Sample size calculation

The primary objective of this study was to examine maternal complications associated with spontaneous vaginal delivery. Data obtained from the Department of Obstetrics and Gynaecology at Siriraj Hospital indicated that the overall prevalence of maternal complications linked to assisted vaginal delivery stands at approximately 10%, with a 95% confidence interval that falls comfortably within an acceptable margin of error of 1%. These calculations were meticulously executed using the nQuery Sample Size Software, utilizing the normal approximation method for confidence interval estimation. As a result, a calculated sample size of 3,458 was determined for this research endeavor. The researcher plans to gather data spanning a period of two years, culminating in a retrospective analysis of a comprehensive dataset comprising 3,500 cases.

### Statistical analysis

Demographic particulars were synthesised using descriptive statistics. Categorical data are delineated as numbers and percentages, while continuous data are represented as the means ± standard deviations or medians accompanied by their respective ranges. Analytical procedures were executed employing PASW Statistics (version 18.0; SPSS Inc., Chicago, IL, USA). Comparative analyses of baseline data (qualitative variables and maternal and neonatal complications stemming from caesarean sections) were facilitated using the chi-squared test complemented by Fisher’s exact test. For quantitative variables, the Mann–Whitney U test was used for univariate analysis, and multiple logistic regression was employed for multivariate analysis.

## Results

The study included 3500 pregnant women who underwent vaginal deliveries. Their mean age was 28.94 ± 6.09 years, ranging from 14 to 48 years. Just over half were experiencing their second or subsequent pregnancies (52.5%; 837 cases). Delivery typically occurred at or beyond 37 weeks of gestation (97.0%; 3396 cases). Most women had haematocrit levels above 33% (Table 1).

**Table 1:** Maternal demographics, pertinent laboratory data and underlying diseases

Data	n = 3500
Maternal age (y)	28.94 ± 6.09 (14, 48)
Body weight (kg)	68.77 ± 11.64 (41.80, 133.30)
BMI (kg/m^2^)	27.18 ± 4.21 (16.96, 49.26)
Parity	
0 ≥ 1	1663 (47.5%) 1837 (52.5%)
Gestational age at delivery (wk)	
< 37 ≥ 37	104 (3.0%) 3396 (97.0%)
Number of antenatal care visits	
0–4 > 4	255 (7.3%) 3245 (92.7%)
Haemoglobinopathy	
Normal Hb H disease Beta thalassaemia Hb E trait Alpha thalassaemia Other	2459 (70.3%) 8 (0.2%) 46 (1.3%) 620 (17.7%) 203 (5.8%) 164 (4.7%)
HBs antigen	
Negative Positive	3414 (97.5%) 86 (2.5%)
Anti-HIV	
Non-reactive Reactive	3481 (99.5%) 19 (0.5%)
Haematocrit	
≤ 33% > 33% Missing data	622 (17.8%) 2831 (80.9%) 47 (1.3%)
Maternal underlying disease	
Heart disease Thyroid disease Pulmonary disease Myoma uteri Ovarian tumours/cysts Others	27 (0.8%) 72 (2.1%) 6 (0.2%) 56 (1.6%) 9 (0.3%) 3 (0.13%)

Of the women, 93.5% (3271 out of 3500) had spontaneous vaginal births, 6.0% (211 out of 3500) underwent vacuum-assisted delivery, and 0.3% (12 out of 3500) had forceps-assisted delivery. Most deliveries (50.7%; 1775 out of 3,500) were performed by trainee physicians, with 63.3% (2214) of the 3500 deliveries occurring outside the standard office hours of 9 AM to 4 PM. Cervical injuries were noted in 3.7% (129 out of 3500) of the women, and 68.4% (2395 out of 3500) were admitted during the active phase of labour (less than 6 hours). The most frequent complication observed was postpartum haemorrhage, affecting 7.3% (256 out of 3,500; Table 2).

**Table 2:** Detailed information regarding vaginal deliveries

Relevant information		n = 3500
Type of vaginal delivery		
Vaginal delivery Vacuum-assisted extraction Forceps-assisted extraction Breech delivery		3271 (93.5%) 211 (6.0%) 12 (0.3%) 6 (0.2%)
**Surgeon (can have more than 1 surgeon)**		
• Resident - *First year* - *Second year* - *Third year* • Fellowship • Staff - *30 to < 40 years* - *40 to < 50 years* - *≥ 50 years* • Resident and staff • Medical students • Nurses		1775 (50.7%) 844 (24.1%) 462 (13.2%) 2 (0.1%) 59 (1.7%) 54 (1.5%) 99 (2.8%) 77 (2.2%) 50 (1.4%) 155 (4.4%)
**Timing**		
• Office hours (9.00 AM–4.00 PM) • Outside office hours (4.00 PM–9.00 AM)		1286 (36.7%) 2214 (63.3%)
**Anaesthesia**		
Local Spinal Epidural Combined spinal and epidural anaesthesia None		3349 (95.7%) 3 (0.1%) 55 (1.6%) 51 (1.5%) 42 (1.1%)
**Duration of active phase of labour (hours)**		
< 6 6 to < 12 12 to < 18 18 to < 24 ≥ 24 Birth before admission N/A		2395 (68.4%) 486 (13.9%) 33 (0.9%) 12 (0.3%) 3 (0.1%) 59 (1.8%) 512 (14.6%)
**Degree of perineal tear**		
No tear First degree Second degree Third degree Fourth degree Abrasion		38 (1.1%) 112 (3.2%) 3104 (88.7%) 223 (6.4%) 11 (0.3%) 12 (0.3%)
**Maternal complications** (N = 449 [12.8%])		n (%)
Post-partum haemorrhage Cervical injury Vaginal injury Labia injury Puerperal infection Other		256 (7.3%) 129 (3.7%) 77 (2.2%) 61 (1.7%) 7 (0.2%) 37 (1.1%)

* Data are presented as mean ± SD (range) and n (%)

Forceps-assisted vaginal delivery resulted in maternal complications in 33.33% of cases (4 out of 12 cases), while vacuum extraction was implicated in 18.96% (40 out of 221). All maternal complications were 12.83% (449 out of 3500 cases); *P* = 0.004). Postpartum haemorrhage was commonly associated with forceps-assisted delivery (33.33%; 4 out of 12) and vacuum-assisted delivery (12.80%; 27 out of 221; *P* < 0.001; Table 3).

**Table 3:** Correlation between maternal complications, vaginal delivery method and gestational age at delivery (univariate analysis)

Complications	All (N = 3500)	Vaginal delivery n (%)				*P*	Gestational age at delivery (wk) n (%)		*P*
		Spontaneous (n = 3271)	Vacuum extraction (n = 221)	Forceps assisting/ extraction (n = 12)	Breech delivery (n = 6)		< 37 (n = 104)	≥ 37 (n = 3396)	
Post-partum haemorrhage	256 (7.31%)	225 (6.88%)	27 (12.80%)	4 (33.33%)	0 (0.00%)	< 0.001^*^	6 (5.77%)	250 (7.36%)	0.539
Puerperal infection	7 (0.20%)	5 (0.15%)	2 (0.95%)	0 (0.00%)	0 (0.00%)	0.097	0 (0.00%)	7 (0.21%)	1.000
Cervical injury	129 (3.69%)	118 (3.61%)	11 (5.21%)	0 (0.00%)	0 (0.00%)	0.545	2 (1.92%)	127 (3.74%)	0.591
Vaginal passage injury	77 (2.20%)	70 (2.14%)	7 (3.32%)	0 (0.00%)	0 (0.00%)	0.640	2 (1.92%)	75 (2.21%)	1.000
Labial injury	61 (1.74%)	59 (1.80%)	2 (0.95%)	0 (0.00%)	0 (0.00%)	0.761	3 (2.88%)	58 (1.71%)	0.428
Rectal, ligament or pelvic joint injury	37 (1.06%)	29 (0.89%)	8 (3.79%)	0 (0.00%)	0 (0.00%)	0.001^*^	1 (0.96%)	36 (1.06%)	1.000
**All maternal complications**	**449 (12.83%)**	**405 (12.38%)**	**40 (18.96%)**	**4 (33.33%)**	**0 (0.00%)**	**0.004** ^*^	**13 (12.50%)**	**436 (12.84%)**	**0.919**

In the univariate analysis, all maternal complications, with notable emphasis on postpartum haemorrhage showed significant associations with a prolonged second stage of labour (18.81%; 19 out of 101 cases; *P* < 0.001), foetal distress (13.10%; 19 out of 145; *P* = 0.006), neonates categorised as large for their gestational age (25.00%; 7 out of 28; *P* = 0.001) and elevated maternal weight (averaging 71.4 ± 12.2 kg; *P* < 0.001). Puerperal infections were significantly linked with a prolonged second stage of labour (1.98%; 2 out of 101; *P* = 0.016) and anaemic women (haematocrit levels < 33%; 0.80%; 5 out of 622; *P* = 0.003). Cervical injuries had a significant association with neonates categorised as large for their gestational age (weighing ≥ 4000 g; 10.71%; 3 out of 28 cases; *P* = 0.035; Table 4).

**Table 4:** Correlations between maternal complications arising from vaginal delivery and potential influencing factors (univariate analysis)

	All (N = 3500)	Maternal underlying disease		*P*	Prolonged second stage of labour		*P*	Foetal distress				*P*		
		No (n = 3399)	Yes (n = 101)		No (n = 3399)	Yes (n = 101)		No (n = 3355)		Yes (n = 145)				
Post-partum haemorrhage	256 (7.31%)	245 (7.21%)	11 (10.89%)	0.161	237 (6.97%)	19 (18.81%)	< 0.001	237 (7.06%)		19 (13.10%)		0.006		
Puerperal infection	7 (0.20%)	6 (0.18%)	1 (0.99%)	0.185	5 (0.15%)	2 (1.98%)	0.016	6 (0.18%)		1 (0.69%)		0.257		
Cervical injury	129 (3.69%)	125 (3.68%)	4 (3.96%)	0.788	122 (3.59%)	7 (6.93%)	0.098	121 (3.61%)		8 (5.52%)		0.232		
Vaginal birth passage injury	77 (2.20%)	72 (2.12%)	5 (4.95%)	0.070	75 (2.21%)	2 (1.98%)	1.000	73 (2.18%)		4 (2.76%)		0.560		
Labial injury	61 (1.74%)	60 (1.77%)	1 (0.99%)	1.000	60 (1.77%)	1 (0.99%)	1.000	60 (1.79%)		1 (0.69%)		0.518		
Other	37 (1.06%)	36 (1.06%)	1 (0.99%)	1.000	34 (1.00%)	3 (2.97%)	0.089	32 (0.95%)		5 (3.45%)		0.017		
**All maternal complications**	**449 (12.83%)**	**432 (12.71%)**	**17 (16.83%)**	**0.222**	**424** **(12.47%)**	**25** **(24.75%)**	**< 0.001**	**421** **(12.55%)**		**28** **(19.31%)**		**0.017**		
	**All** **(N = 3500)**	**Maternal haematocrit**		***P***	**Neonatal birthweight (g)**			***P***	**Maternal weight (kg)**				***P***	
		**Hct ≤ 33%** **(n = 622)**	**Hct > 33%** **(n = 2831)**		AGA* **(2500–4000)** **(n = 3233)**	SGA** **(1500–2499)** **(n = 239)**	LGA*** **(> 4000)** **(n = 28)**		**No**		**Yes**			
Post-partum haemorrhage	256 (7.31%)	49 (7.88%)	205 (7.24%)	0.582	235 (7.27%)	14 (5.86%)	7 (25.00%)	0.001	68.6 ± 11.6		71.4 ± 12.2		< 0.001	
Puerperal infection	7 (0.20%)	5 (0.80%)	2 (0.07%)	0.003	7 (0.22%)	0 (0.00%)	0 (0.00%)	0.749	68.8 ± 11.6		71.6 ± 12.8		0.519	
Cervical injury	129 (3.69%)	22 (3.54%)	106 (3.74%)	0.804	122 (3.77%)	4 (1.67%)	3 (10.71%)	0.035	68.8 ± 11.7		68.3 ± 10.5		0.614	
Vaginal birth passage injury	77 (2.20%)	14 (2.25%)	62 (2.19%)	0.925	69 (2.13%)	7 (2.93%)	1 (3.57%)	0.638	68.7 ± 11.7		69.9 ± 10.4		0.367	
Labial injury	61 (1.74%)	10 (1.61%)	49 (1.73%)	0.830	51 (1.58%)	9 (3.77%)	1 (3.57%)	0.034	69.2 ± 13.1		68.8 ± 11.6		0.763	
Other	37 (1.06%)	6 (0.96%)	30 (1.06%)	0.833	32 (0.99%)	4 (1.67%)	1 (3.57%)	0.259	68.8 ± 11.6		69.7 ± 13.6		0.609	
**All maternal complications**	**449** **(12.83%)**	**89** **(14.31%)**	**353** **(12.47%)**	**0.214**	**414** **(12.81%)**	**27** **(11.30%)**	**8** **(28.57%)**	**0.035**	**68.6 ± 11.0**		**670.0 ± 12.0**		**0.013**	

*AGA, appropriate gestational age; ***LGA, large for gestational age; **SGA, small for gestational age

Multivariate analysis revealed that postpartum haemorrhage was significantly associated with gestational diabetes mellitus class A1 (adjusted odds ratio [AOR] 1.46; 95% confidence interval [CI] 1.01–2.11; *P* = 0.045), forceps-assisted delivery (AOR 5.11; 95% CI 1.30–20.1; *P* = 0.020), prolonged second stage of labour (AOR 2.68; 95% CI 1.09–6.58; *P* = 0.032), elevated maternal overweight (averaging 71.4 ± 12.2 kg; AOR 1.02; 95% CI 1.01–1.03; *P* = 0.003) and neonates identified as large for their gestational age (AOR 3.02; 95% CI 1.23–7.43; *P* = 0.016; Table 5). Postpartum haemorrhage was not related to the surgeons who performed delivery (adjusted odds ratio [AOR] 0.58; 95% confidence interval [CI] 0.28–1.20; *P* = 0.144) (Table 5).

**Table 5:** Factors correlated with postpartum haemorrhage (multivariable analysis)

Associated factors (n = 256)	Unadjusted odds ratio (95% CI)	*P*	Adjusted odds ratio (95% CI)	*P*
Gestational diabetes mellitus				
No Class A1 Class A2 Pre-existing diabetes mellitus	Ref. 1.51 (1.05, 2.17) 1.42 (0.33, 6.13) –	0.026 0.639 –	Ref. 1.46 (1.01, 2.11) 1.69 (0.38, 7.45) –	0.045^*^ 0.491 –
Hypertension (HT)				
No Gestational HT/transient Severe pre-eclampsia Pre-existing HT	Ref. 1.54 (0.96, 2.47) 1.86 (0.42, 8.21) –	0.072 0.415 –	Ref. 1.28 (0.78, 2.09) 1.62 (0.35, 7.46) –	0.325 0.533 –
Surgeon				
Resident and Fellow Staff Medical students and midwives	Ref. 0.50 (0.24, 1.03) 1.01 (0.59, 1.70)	0.058 0.991	Ref. 0.58 (0.28, 1.20) 0.90 (0.52, 1.57)	0.144 0.720
Vaginal delivery				
Spontaneous Vacuum-assisted extraction Forceps-assisted extraction Breech delivery	Ref. 1.99 (1.30, 3.04) 6.77 (2.02, 22.7) –	0.002 0.002 –	Ref. 0.89 (0.32, 2.47) 5.11 (1.30, 20.1) –	0.821 0.020^*^ –
Underlying disease	1.57 (0.83, 2.98)	0.161	1.65 (0.86, 3.17)	0.136
Prolonged second stage of labour	3.09 (1.85, 5.18)	< 0.001	2.68 (1.09, 6.58)	0.032^*^
Foetal distress	1.98 (1.20, 3.27)	0.006	1.32 (0.55, 3.16)	0.539
High maternal weight (71.4 ± 12.2 kg)	1.02 (1.01, 1.03)	< 0.001	1.02 (1.01, 1.03)	0.003^*^
Neonatal birthweight (g)				
Normal (2500–4000) Low 1500–2499) High (> 4000)	Ref. 0.79 (0.46, 1.38) 4.25 (1.79, 10.1)	0.416 0.001	Ref. 0.90 (0.51, 1.59) 3.02 (1.23, 7.43)	0.721 0.016^*^

## Discussion

Approximately half of the 3500 pregnant women who underwent vaginal delivery at Siriraj Hospital between 2020 and 2022 were multiparous (52.5%; 1,837 out of 3500). The vast majority delivered at term (gestational age ≥ 37 weeks; 97.0%; 3396 out of 3,500), and a substantial proportion were of advanced maternal age (≥ 35 years; 34.14%; 1195 out of 3500).

Assisted vaginal delivery, performed using forceps or a vacuum, is a standard obstetric procedure. Its primary aim is to accelerate the birthing process, facilitating the prompt delivery of the baby. Complications linked to these procedures can stem from a range of factors, often interrelated. These factors encompass the choice of equipment, the baby’s head position during the procedure, the specific indications for the intervention, and, critically, the experience of the attending physician. Furthermore, other risk factors, such as the duration of the second stage of labor, also play a significant role [8].

Assisted vaginal delivery can lead to maternal complications, including lower genital tract lacerations, vulvar and vaginal hematomas, urinary tract injury, and anal sphincter injury [8–11]. The risk of maternal trauma, especially third and fourth-degree perineal lacerations, is higher when the baby is in an occiput posterior position during assisted vaginal delivery [12–14]. The complexity of the instrumental intervention is directly related to increasing maternal morbidity, with spontaneous vaginal birth being the least morbid, followed by vacuum extraction, forceps-assisted birth, and caesarean birth, which carries concerns about venous thromboembolism, endometritis, and wound infection. Surprisingly, caesarean delivery, despite protecting the genital tract from forceps/vacuum-related trauma, does not lower the long-term risk of urinary incontinence, anal incontinence, and prolapse symptoms when compared to second-stage assisted vaginal birth in longitudinal cohort studies [15, 16].

In our study, assisted forceps deliveries were found to be associated with injuries to the vaginal birth passage, while neonates classified as large-for-gestational-age showed a significant correlation with cervical and labial injuries. It’s important to highlight that injuries to the mother’s pelvic floor, a muscle group that influences the urinary, genital, and gastrointestinal systems located at the base of the pelvis, were frequently observed following assisted forceps deliveries. Recent advancements in the practice of assisted forceps delivery have undergone significant evolution, primarily aimed at mitigating these complications [17].

Postpartum haemorrhage emerged as the predominant maternal complication, with an incidence of 7.31% among the cases (256 out of 3500). This complication exhibited a significant association with spontaneous vaginal deliveries (6.88%; 225 out of 3271) and assisted vaginal deliveries, encompassing forceps-assisted births (33.33%; 4 out of 12 cases) and vacuum extractions (12.80%; 27 out of 221; P < 0.0001). Our findings highlight that forceps-assisted deliveries are linked to a higher rate of maternal complications compared to vacuum-assisted deliveries, a conclusion supported by the study conducted by Kabiru et al. [18].

Our analysis has also emphasized that an extended second stage of labor represents a significant risk factor for postpartum haemorrhage. This conclusion finds support in a previous study that identified a prolonged second stage of labor as a risk factor for postpartum haemorrhage [19]. Competent physicians can adeptly and safely perform forceps and vacuum interventions when appropriately indicated. The American College of Obstetricians and Gynecologists, in conjunction with the Society for Maternal-Fetal Medicine, recommends allowing up to 3 hours of pushing for nulliparous patients and up to 2 hours for multiparous women before diagnosing an arrest of labor [20]. This approach aims to reduce the need for caesarean sections resulting from second-stage progression failure. However, adopting these diagnostic criteria may inadvertently precipitate postpartum haemorrhage following assisted vaginal deliveries. A prolonged second stage invariably leads to uterine myofiber edema, brittleness, thinning of the uterus’s lower segment, and poor uterine contractions, all of which amplify blood loss. Consequently, the choice of interventions in cases of prolonged delivery time may be associated with an increased risk of postpartum haemorrhage.

The practice bulletin from the American College of Obstetricians and Gynecologists [21] outlines the recommended indications for assisted vaginal delivery, which include maternal exhaustion, underlying maternal medical conditions (e.g., cardiac disease), a prolonged second stage of labor, and foetal compromise. These situations can occur when the patient is not adequately equipped to push effectively or when there is an emergent, life-threatening foetal condition.

The decision to proceed with assisted vaginal delivery is typically made when a clinician has confidence in its potential success, although certainty is never guaranteed. A failed attempt can result in injuries to the birth canal and may trigger postpartum haemorrhage [22, 23]. The decision-making process for assisted vaginal birth is dynamic, with ongoing reassessments being conducted based on the outcomes of each step. Unfortunately, preoperative risk factors cannot consistently predict the outcome of a vaginal delivery attempt [24].

In our study, the rates of vacuum- and forceps-assisted vaginal deliveries were 6.3% and 0.3%, respectively. Both forceps and vacuum-assisted deliveries were relatively rare in comparison to spontaneous vaginal deliveries. This discovery is consistent with the findings of a substantial prospective study carried out in low- and middle-income countries. The study reported a decrease in assisted vaginal delivery rates from 1.6 to 0.3% between 2010 and 2016, while the rates of caesarean sections more than doubled, reaching 14.4% [25].

Limited occurrences of operative deliveries can hinder resident training, and the increasing preference for caesarean sections might exacerbate this situation. A significant challenge for future obstetricians will be attaining proficiency in operative deliveries, given the scarcity of such cases. The President of the Royal College of Obstetricians and Gynaecologists should prioritize this as a crucial educational concern for residents [6]. As birth rates decrease, the opportunities for hands-on experience and skill development also diminish, potentially raising risks for both mothers and neonates.

Our study revealed a noteworthy association between puerperal infection and maternal anaemia (haematocrit levels < 33%), a discovery supported by Melkie and Dagnew’s research [26]. Anaemia can undermine the body’s natural defense system, the immune system, leading to an increased susceptibility to infections [27]. It is crucial to address anaemia during pregnancy to mitigate the risk of puerperal infections following delivery.

After accounting for confounding factors, postpartum haemorrhage showed a significant association with gestational diabetes mellitus class A1, assisted forceps delivery, an extended second stage of labor, elevated maternal weight, and foetal macrosomia. Inadequately managed gestational diabetes is linked to the birth of large-for-gestational-age neonates and an increase in maternal weight. The presence of dense, soft tissue in the vaginal canal can hinder the delivery process, leading to a prolonged second stage. Forceps-assisted births may entail a heightened risk of birth canal injuries and subsequent postpartum haemorrhage. However, a previous study reported that women requiring interventions during the second stage, with valid indications for forceps, experienced few maternal infections but had a higher incidence of postpartum haemorrhage [28].

### Strengths of the study

Our study leverages a substantial dataset involving a diverse cohort of patients from a large sample size, a two-year period, offering valuable insights into maternal complications associated with vaginal deliveries. The inclusion of multiple factors influencing maternal complications, from foetal weight and duration of the second stage of labour to the experience of medical professionals, enriches the comprehensiveness of our findings.

### Limitations of the study

Despite the robust dataset, this study is retrospective in nature and is therefore subject to inherent limitations associated with such designs. As with any observational study, causality cannot be definitively.

## Conclusions

The primary complication observed in assisted vaginal delivery was postpartum haemorrhage. Predisposing factors were a prolonged second stage of labour, foetal distress, foetal size large for gestational age and elevated maternal weight. Cervical and labial injuries showed a notable association with large-for-gestational-age neonates. Puerperal infections were linked to maternal anaemia (haematocrit levels < 33%).

### Disclosure of interests

Each author adhered to the protocol prescribed by the International Committee of Medical Journal Editors and completed the Form for Uniform Disclosure of Potential Conflicts of Interest. No conflicts were identified by any of the authors. All protocols utilised in this retrospective chart review were consistent with the ethical standards set by the institutional research committee (Si 060/2020) and the 1964 Declaration of Helsinki, along with its subsequent amendments or equivalent ethical guidelines. This was a retrospective study without experiments on humans and/or the use of human tissue samples. The institutional and/or licensing committee approving the experiments is not available.

## Data Availability

The datasets used or analysed during the current study available from the corresponding author on reasonable request.
